# Plantar load transfer in children: a descriptive study with two pathological case studies

**DOI:** 10.1186/s12891-021-04364-9

**Published:** 2021-06-07

**Authors:** Alexis Brierty, Christopher P. Carty, Claudia Giacomozzi, Teresa Phillips, Henry P. J. Walsh, David Bade, Sean Horan

**Affiliations:** 1 Griffith Centre of Biomedical and Rehabilitation Engineering (GCORE), Menzies Health Institute Queensland, Griffith University, Gold Coast, QLD 4222 Australia; 2 Queensland Children’s Motion Analysis Service, Queensland Children’s Hospital, Brisbane, QLD 4101 Australia; 3 School of Allied Health Sciences, Griffith University, Gold Coast, QLD 4222 Australia; 4 Department of Orthopaedic Surgery, Queensland Children’s Hospital, Brisbane, QLD 4101 Australia; 5 Research Development Unit, Caboolture Hospital, Metro North Hospital and Health Service, Caboolture, Gold Coast, QLD 4510 Australia; 6 Italian National Institute of Health (Istituto Superiore di Sanità), Viale Regina Elena, 299, 00161 Rome, RM Italy

**Keywords:** Plantar pressure, Plantar load, Load transfer, Typically developed, Children

## Abstract

**Background:**

Typical gait is often considered to be highly symmetrical, with gait asymmetries typically associated with pathological gait. Whilst gait symmetry is often expressed in symmetry ratios, measures of symmetry do not provide insight into how these asymmetries affect gait variables. To fully understand changes caused by gait asymmetry, we must first develop a normative database for comparison. Therefore, the aim of this study was to describe normative reference values of regional plantar load and present comparisons with two pathological case studies.

**Methods:**

A descriptive study of the load transfer of plantar pressures in typically developed children was conducted to develop a baseline for comparison of the effects of gait asymmetry in paediatric clinical populations. Plantar load and 3D kinematic data was collected for 17 typically developed participants with a mean age of 9.4 ± 4.0 years. Two case studies were also included; a 10-year-old male with clubfoot and an 8-year-old female with a flatfoot deformity. Data was analysed using a kinematics-pressure integration technique for anatomical masking into 5 regions of interest; medial and lateral forefoot, midfoot, and medial and lateral hindfoot.

**Results:**

Clear differences between the two case studies and the typical dataset were seen for the load transfer phase of gait. For case study one, lateral bias was seen in the forefoot of the trailing foot across all variables, as well as increases in contact area, force and mean pressure in the lateral hindfoot of the leading foot. For case study two, the forefoot of the trailing foot produced results very similar to the typical dataset across all variables. In the hindfoot of the leading foot, medial bias presents most notably in the force and mean pressure graphs.

**Conclusions:**

This study highlights the clinical significance of the load transfer phase of gait, providing meaningful information for intervention planning.

**Supplementary Information:**

The online version contains supplementary material available at 10.1186/s12891-021-04364-9.

## Background

Plantar pressure measurement provides insight into the foot-ground surface interaction during the stance phase of gait and is commonly used to describe specific load characteristics at the sole of the foot [1]. Plantar pressures have been used to inform clinical management for numerous patient populations including individuals with diabetes [2, 3], cerebral palsy (CP) [4, 5] and congenital talipes equinovarus (CTEV) [6, 7]. Platform systems used to conduct plantar pressure assessments typically comprise a flat rigid array of sensors, allowing the capture of barefoot gait across a ground-like surface [8]. Depending on the size of the platform, this can include one or multiple steps in a single pass. The most commonly reported parameters include contact area, contact time, peak pressure, mean pressure, pressure-time-integral, force and force-time-integrals [6, 9–14]. Each parameter can provide the clinician with valuable information to determine the best course of action and prevent serious complications that can have a major effect on a patient’s quality of life.

When interpreting plantar pressure data and making clinical decisions in paediatric populations, it is essential to have an age matched reference data set [10, 12], as well as a clear understanding of foot kinematics in paediatric population [15, 16]. In a typically developing child that has achieved a mature gait pattern, stance during barefoot walking is initiated by hindfoot contact with a slightly inverted calcaneus, which everts from initial contact through to weightbearing, followed by a lateral to medial transition of the forefoot contact to the ground necessary for stabilisation of the first metatarsal for push off [16]. This results in plantar pressures that are initially located at the centre of the heel and progress laterally through the midfoot towards the second and third metatarsal heads and finally to hallux [14]. Plantar pressure patterns can be altered by factors such as strike patterns [11], walking speed [14], joint mobility [9, 14], age [10, 12, 17] and gender [17–19]. However, despite these variations, typically developing children (aged 7–11) are considered to demonstrate reliable and repeatable patterns across all regions of the foot, with the exception of the lesser toes [13]. Analysis of plantar variables across the different regions of the foot is achieved through subsampling; the most common method of analysis of plantar load data [20].

Subsampling, also known as masking, is guided by footprint morphology and facilitates an objective and clinically relevant analysis compared to examining the whole foot [6]. There is, however, a lack of standardisation in how the foot is divided into regions of interest (ROIs), making comparisons between studies difficult. Masking methods can be manual or automated, with manual masking relying on visual detection of anatomical landmarks [12] and therefore prone to human error. In contrast, geometric masking is an automated method and divides the footprint into segments using algorithms based on typical plantar pressure maps [10]. Whilst geometric masking reduces the impact of user error, it can be unsuccessful when assessing abnormal or incomplete footprints with poor foot-to-ground interaction [21]. Anatomical masking, another automated method, uses anatomical landmarks to divide the footprints into ROIs based on data from alternate measurement devices, including x-rays, tomography, MRI scans, physical markers placed under the sole of the foot or 3D marker models [21–23]. Anatomical masking using 3D motion analysis systems is a relatively novel approach that allows the integration of pressure, force, and kinematic data. It was first described by Giacomozzi and colleagues [21], and has since been validated in typically developing children and multiple paediatric populations [1, 24, 25]. The method can be used to divide the foot into 5 or more ROI’s depending on the foot model used, and has been shown to be more sensitive in detecting differences between healthy and pathological populations when compared to geometric masking [6].

Many plantar pressure measurements, including contact area, forces, and pressures, are univariate in that they are collected from isolated individual steps. It is therefore assumed that the gait of the participants in studies that collect data from an isolated step is either symmetrical or unaffected by the opposing limb. Hannah and colleagues [26] suggest that the assumption of symmetry has led to the development and widespread use of measurement systems designed to record kinematic, kinetic or temporal variables of a single limb. Despite this, typical gait is not perfectly symmetrical and repetitive, and therefore small and random asymmetries within a standard deviation are considered normal [27]. Gait symmetry is often measured by calculating the differences between the left and right side for a given parameter [28], and producing symmetry indices or ratios. Gait parameters in typical gait usually produce low symmetry ratios, suggesting highly symmetrical gait patterns. When asymmetries are present, it is often symptomatic of pathological gait [28]. Gait symmetry has been used to identify pathologies in gait and to track recovery [28] by examining parameters including ground reaction forces (GRF), electromyography (EMG), plantar pressure and temporal-spatial parameters [29]. Symmetry indices, however, only calculate the magnitude of the difference, whereas integrated pressure-kinematic data may allow the clinician to isolate a specific region of interest that will benefit from a targeted clinical intervention.

It is clear from reviewing the literature that little is known about how gait asymmetries affect regional foot pressures during the transfer phase of gait in paediatric clinical populations. An important first step in being able to achieve this is to first describe typical paediatric gait during the load transfer phase. Therefore, the primary aim of this study was to describe normative reference values of regional plantar pressures during the load transfer phase of gait. The secondary aim was to present two pathological case studies with abnormal joint morphology and mobility, and discuss relative to the reference data, in an effort to outline the clinical utility of plantar pressure measurement when used in conjunction with anatomical masking.

## Methods

### Sample information

Seventeen typically developed (TD) children with no history of major lower limb injury or physical disability were recruited for this study. The cohort consisted of 9 males and 8 females with a mean age of 9.4 ± 4.0 years. Participants had a mean height of 135.8 ± 21.9 cm and mass of 31.7 ± 14.5 kg.

Two clinical participants that were referred for clinical gait analysis for foot impairment were also included as case studies. Case study one was a 10-year-old male with bilateral CTEV. CTEV, commonly referred to as clubfoot, is a rigid deformity characterised by increased equinus, varus and forefoot adduction. Case study one had a height of 147 cm and mass of 58.2 kg. He was previously treated with the Ponseti method, a serial casting protocol used to correct clubfoot [30], and was being considered for tendon transfer surgery at the time of testing. Case study two was an 8-year-old female with bilateral pes planus, or flatfoot. Flatfoot can be either rigid or flexible and is characterised by a low medial longitudinal arch [31]. Case study two had a height of 136 cm and mass of 30.8 kg. She had no previous lower limb surgery prior to data collection.

Informed consent for data collection and publication was collected from a parent and/or legal guardian for all TD and case study participants. Data collection procedures were approved by the institutional Human Research Ethics Committee and performed in accordance with standard operating procedures in the Queensland Children’s Motion Analysis Service (QCMAS).

### Data collection

All participants were required to attend a single data collection session at the QCMAS, with a parent and/or guardian present. Participants were encouraged to bring a drink and snacks to the session and were permitted multiple breaks if necessary. Before markers were attached, several measurements were taken by an experienced physiotherapist, including height, mass, leg length, knee and ankle width, and frontal plane knee alignment. A total of 41 reflective markers (9 mm) were then attached to each participants trunk, pelvis and lower limbs in accordance with the Plug in Gait (PiG) [32] and Oxford Foot Model (OFM) [33] marker sets. Once all markers were attached, each participant stood in the centre of the room in the anatomical position where a static trial was captured. The static trial was then used to perform a knee varus/valgus check as described by Kainz and colleagues [34]. Once both the physiotherapist and biomechanist agreed on the accuracy of the marker placement, walking trials were collected.

For the walking trials, participants were asked to walk across the 1.5 m Novel EMED XL platform (88 × 188 capacitive sensors (4sensors/cm2), novel GmbH, Munich, Germany) at a normal self-selected walking speed. Participants were asked to start behind one of two coloured lines on the ground at the end of the walkway and multiple steps were collected with each pass. Walking trials were collected until the participant had achieved their normal walking patterns, with early trials excluded from analysis. A minimum of 5 transfers for each leg were collected in accordance with suggested protocols by Benedetti and colleagues [35]. The walkway was defined in space using four additional reflective markers, which were attached to the walkway with the centre of the markers at each corner of the collection mat [9]. Marker trajectories were simultaneously collected using a ten-camera, three-dimensional motion capture system (10x Vicon V16 Vantage cameras; Vicon, Oxford, UK). Synchronised capturing of the two data sets was achieved using a trigger system, with all data being collected at 100 Hz.

### Data integration

Plantar pressure data was output as *.lst files using the Novel emascii software. Marker labelling and processing was performed using Vicon Nexus (Version 2.8; Vicon, Oxford, UK), with all marker trajectories output as *.xls files. The positions of the markers were then superimposed onto the pressure footprint [36] using dedicated MATLAB code (R2015a; The Mathworks, Natick, USA). The footprint was divided into 5 ROIs as reported by Stebbins and colleagues [36] including: medial (ROI1) and lateral hindfoot (ROI2), midfoot (ROI3), and medial (ROI4) and lateral forefoot (ROI5). This method was first described by Giacomozzi and colleagues [21] and has been successfully applied using the OFM for the investigation of paediatric clubfoot [1], albeit only for isolated steps. The procedure to optimize the matching between marker configuration at midstance and maximum pressure footprint was further developed from previous studies [1, 14], by taking into account the minimum marker motion along the three axes.

### Data analysis

Plantar load data for the individual regions was output as *.xls spreadsheets. Load transfer (LT) data was then isolated and defined as a left to right (LR) or right to left (RL) transfer. For TD participants, both LR and RL transfers were used. For the case studies, only transfers from least affect to most affected limb were used. We defined the LT phase as time within stance when both feet are in contact with the ground. A dedicated MATLAB code was used to calculate means and standard deviations, as well as time normalise all data. A MATLAB code was also used to produce graphical representations for each participant for peak pressure, mean pressure, vertical force and contact area for each relevant ROI. For the purpose of this study, only regional data was analysed. A minimum of 5 transfers for each participant were used for data analysis purposes.

## Results

**Fig. 1:**
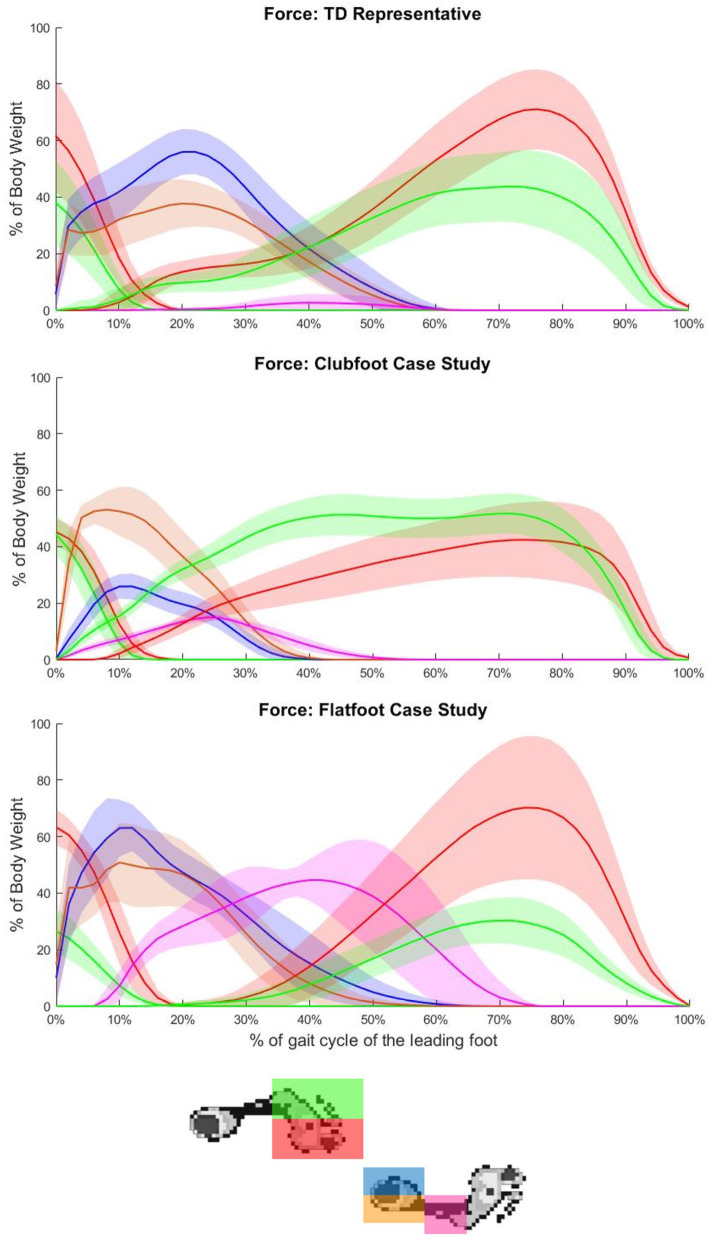
Normalised force means (solid line) and standard deviations (coloured bands) for all regions across the gait cycle of the leading foot for a representative TD participant (1**A**), case study one (1**B**) and case study two (1**C**)

Data was output for all participants aged 4.47 to 16.6 for key variables during the load transfer phase of gait (Supplementary Figures 1 and 2). We have defined the LT phase as the period during which both feet are in contact with the ground (i.e. double support). During typical gait, this will occur from the foot strike of the leading step until the foot off of the trailing step. This phase occurred for the first 8.5% (± 1.3; Table 1) of the leading foot’s stance phase in the typical participants (Fig. 1A). The clubfoot and flatfoot case studies spent the first 13.1% (± 0.8) and 10.1% (± 0.86) respectively (Table 1) of the leading foot’s stance phase in double limb support (Fig. 1B and C). Subsequent results focus predominantly on the LT phase, although relevant information can be acquired from the remainder of stance phase for the leading foot. All trials were time normalised for comparison.

**Table 1:** Temporal spatial data for the two case studies compared to the typically developed population

	Stride Length (m)	Step Width (m)	Velocity (m/s)	% stance time in double limb support
**Typically Developed**	1.08 ± 0.20	0.14 ± 0.03	1.16 ± 0.16	8.45 ± 1.33
**Clubfoot Case Study**	1.16 ± 0.05	0.29 ± 0.02	1.19 ± 0.06	13.1 ± 0.8
**Flatfoot Case Study**	1.30 ± 0.03	0.13 ± 0.03	1.42 ± 0.04	10.08 ± 0.86

### Contact area

**Fig. 2:**
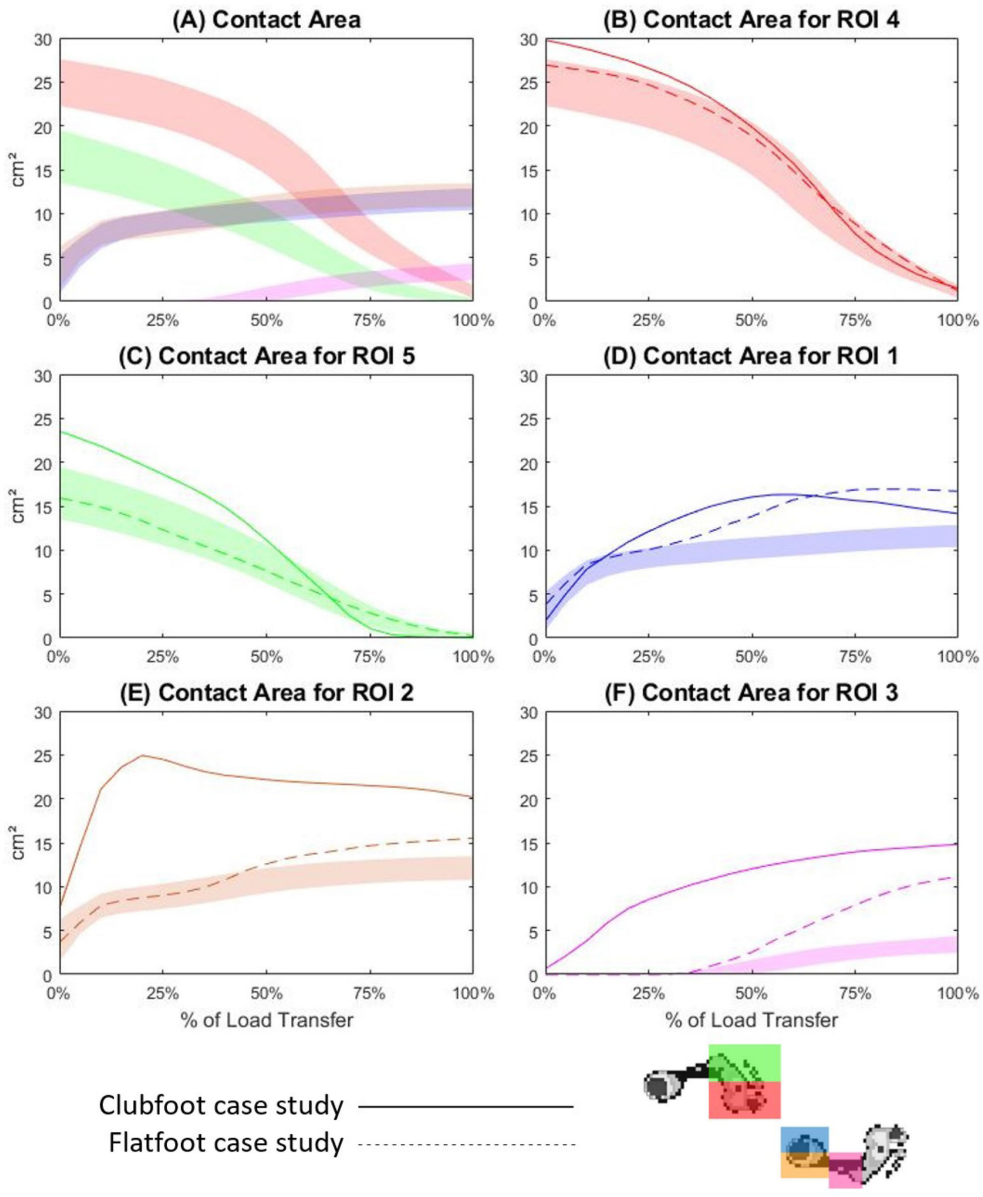
Contact area across the load transfer phase of gait for all regions (2**A**), ROI4 (2**B**), ROI5 (2**C**), ROI1 (2**D**), ROI2 (2**E**) and ROI3 (2**F**). The coloured band is representative of one standard deviation either side of the mean for the TD cohort, the solid line is the mean trace for case study one, and the dotted line is the mean trace for case study two

In the typical participants, the regions with the largest contact areas at the start of the LT phase were ROI4 and ROI5 with averages of 24.93 cm^2^ and 16.52 cm^2^ respectively (Fig. 2A, B and C). Both regions exhibited a steady decline to < 2 cm^2^ across LT. Contact area in ROI1 and ROI2 averaged 3.46 cm^2^, before a sharp incline to an average of 8.31 cm^2^ at 15% of the LT phase (Fig. 2A, D and E). Contact area in these regions then increased by an average of 4.85 cm^2^ across the remainder of LT. ROI3 did not exceed 0.2 cm^2^ until 35% of LT and rose steadily to an average of 3.41 cm^2^ at the completion of the LT phase (Fig. 2A and F). For the clubfoot case study, contact areas in ROI4 and ROI5 both increased at the commencement of LT (Supplementary Table 1), and exhibited similar patterns across the remaining LT phase, overlapping with the TD band at 45 and 55% respectively (Fig. 2B and C). For ROI1, case study one contact area mean initially overlapped the TD band, before increasing above the TD upper limit at 20% of the LT phase and peaking at 16.31 cm^2^ at 60% (Fig. 2D). The mean then declined to 14.13 cm^2^ across the remainder of the LT phase. For ROI2, the contact area mean for case study one began above the TD band, sharply increasing to 24.92 cm^2^ at 20% of the LT phase before declining to 20.18 cm^2^ across the remainder of the LT phase (Fig. 2E). Contact area for ROI3 also began above the TD band for case study one, with a steady incline to 14.82 cm^2^ across the LT phase (Fig. 2F). For case study two, mean contact area for ROI4 and ROI5 overlapped the TD band for the entire LT phase (Fig. 2B and C). Case study two contact area for ROI1 showed a similar pattern to the TD band, until increasing above the TD upper limit at 30%, before peaking at 16.54 cm^2^ at 70% of the LT phase (Fig. 2D). Contact area for ROI2 showed a similar pattern, increasing above the TD band at 45% of the LT phase and peaking at 15.52 cm^2^ at 100% (Fig. 2E). Finally, case study two’s mean contact area for ROI3 exhibited a steep incline from 30 to 100% of the LT phase, peaking at 11.19 cm^2^ (Fig. 2F).

### Force

**Fig. 3:**
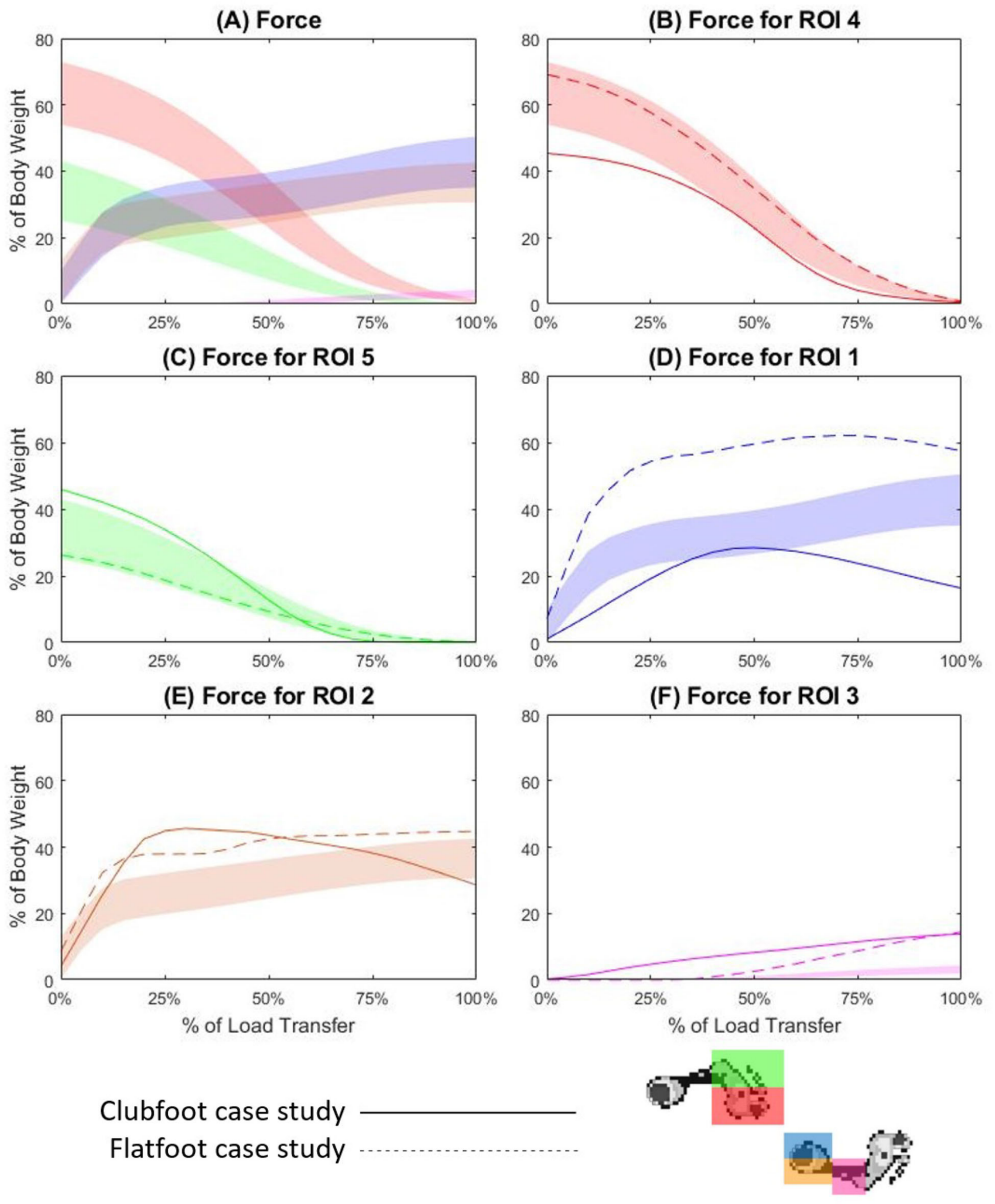
Normalised force across the load transfer phase of gait for all regions (3**A**), ROI4 (3**B**), ROI5 (3**C**), ROI1 (3**D**), ROI2 (3**E**) and ROI3 (3**F**). The coloured band is representative of one standard deviation either side of the mean for the TD cohort, the solid line is the mean trace for case study one, and the dotted line is the mean trace for case study two

Force data was recorded in newtons and normalised to body weight, to permit inter-trial and inter-subject averaging of data [9]. In the TD cohort, forces recorded in ROI4 and ROI5 commenced LT at an average of 63.50 and 34.11% of body weight respectively (Supplementary Table 1) before steadily declining across the LT phase (Fig. 3A, B and C). Forces in ROI1 and ROI2 showed similar outputs, with a sharp incline to an average of 26.35% of body weight within the first 20% of the LT phase, before a steady incline across the rest of the LT phase (Fig. 3A, D and E). Forces in ROI1 showed a small increase in force across the majority of the LT phase. Furthermore, normalised forces first exceeded 1% of body weight at 50% of LT for ROI3 and showed a steady increase until completion of the LT phase (Fig. 3A and F). For the case study one, the mean force in ROI4 was slightly decreased compared to TD at commencement of the LT phase (Supplementary Table 1) but followed a similar pattern (Fig. 2B). Mean force in ROI5 was slightly increased at commencement of the LT phase (Supplementary Table 1), but once again followed a similar pattern, overlapping the TD band at 40% (Fig. 2C). For case study one, the mean force in ROI1 exhibited a bell-shaped pattern below the lower limits of the TD band, overlapping with the TD band from 35 to 55% of the LT phase; peaking at 28.45% of body weight at 50% (Fig. 2D). Mean force in ROI2 exhibited a skewed bell shape, inclining above the TD band to peak at 45.6% body weight at 30% of the LT phase (Supplementary Table 1 and Fig. 2E). Case study one mean force for ROI3 showed a steady incline to 13.82% of body weight across the entire LT phase (Fig. 2F). Case study two force means for ROI4 and ROI5 remained within the TD band for 90 and 100% of the LT phase respectively (Supplementary Table 1 and Fig. 3B and C). The mean force for ROI1 exhibited a steep incline to approximately double the TD mean, before plateauing across the remainder of the LT phase (Fig. 2D). The mean force for ROI2 followed a similar pattern to the TD band, being above the upper limit the majority of the LT phase (Fig. 2E). Finally, case study two mean force for ROI3 increased from 30 to 100% of the LT phase, peaking at 11.19% of body weight (Fig. 2F).

### Peak pressure

**Fig. 4:**
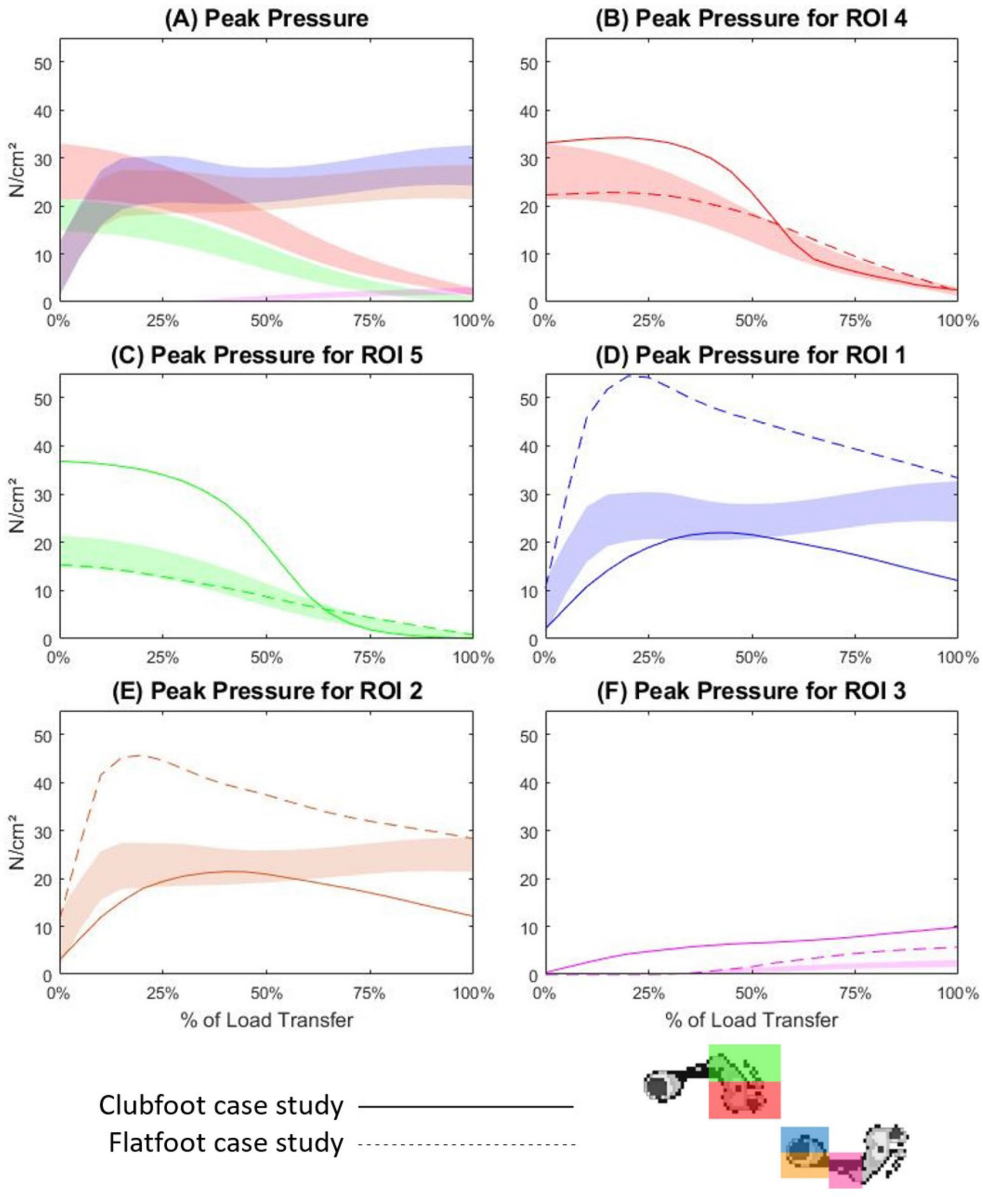
Peak pressure across the load transfer phase of gait for all regions (4**A**), ROI4 (4**B**), ROI5 (4**C**), ROI1 (4**D**), ROI2 (4**E**) and ROI3 (4**F**). The coloured band is representative of one standard deviation either side of the mean for the TD cohort, the solid line is the mean trace for case study one, and the dotted line is the mean trace for case study two

Peak pressures are defined by the single sensor recording the largest pressure within the ROI at each timepoint. At the commencement of LT for the TD participants, the largest peak pressures were recorded at ROIs 4 (27.21 N/cm^2^) and ROI5 (18.15 N/cm^2^) before both steadily decreasing across the remainder of the LT phase (Supplementary Table 1 and Fig. 4A, B and C). Peak pressures in ROI1 and ROI2 showed a steep incline to an average of 23.84 N/cm^2^ at 15% of the LT phase, before plateauing across the remaining part of the LT phase (Fig. 4A, D and E). Peak pressures for ROI1 were greater than those in ROI2 for the majority of the LT phase. Peak pressure values for ROI3 first exceeded 1 N/cm^2^ at 45% of LT and increased steadily to an average of 2.27 N/cm^2^ at the completion of LT. For case study one, average peak pressures were greater than those in the TD population in ROI4 for the first 60% of the LT phase and ROI5 for the first 65%. Peak pressures in both regions showed a rapid decline during the middle 25% of the LT phase, before overlapping the TD band. Case study one peak pressure means for ROI1 and ROI2 both displayed slightly skewed bell-shaped curves below the TD band, peaking within the TD band at 22.02 N/cm^2^ and 21.43 N/cm^2^ respectively (Supplementary Table 1 and Fig. 4D and E). Mean peak pressure for ROI3 showed a steady incline across 100% of the LT phase, with the peak occurring at 9.81 N/cm^2^. For case study two, mean peak pressures for ROI4 and ROI5 were within the TD bands for 65 and 75% of the LT phase respectively (Supplementary Table 1), closely matching the TD band pattern (Figs. 4B and C). Peak pressures in ROI1 exhibited a steep incline to peak at 54.59 N/cm^2^at 20% of the LT phase (Supplementary Table 1) before a moderate decline across the remainder of LT (Fig. 4D). Case study two mean peak pressures for ROI2 exhibited a similar pattern, peaking at 45.68 N/cm^2^ at 20% of the LT phase (Additional file 1 and Fig. 4E). Finally, peak pressures for case study two were first produced in ROI3 at 30% of LT and exhibited a steady increase to 5.66 N/cm^2^ at completion of the LT phase (Supplementary Table 1 and Fig. 4F).

### Mean pressure

**Fig. 5:**
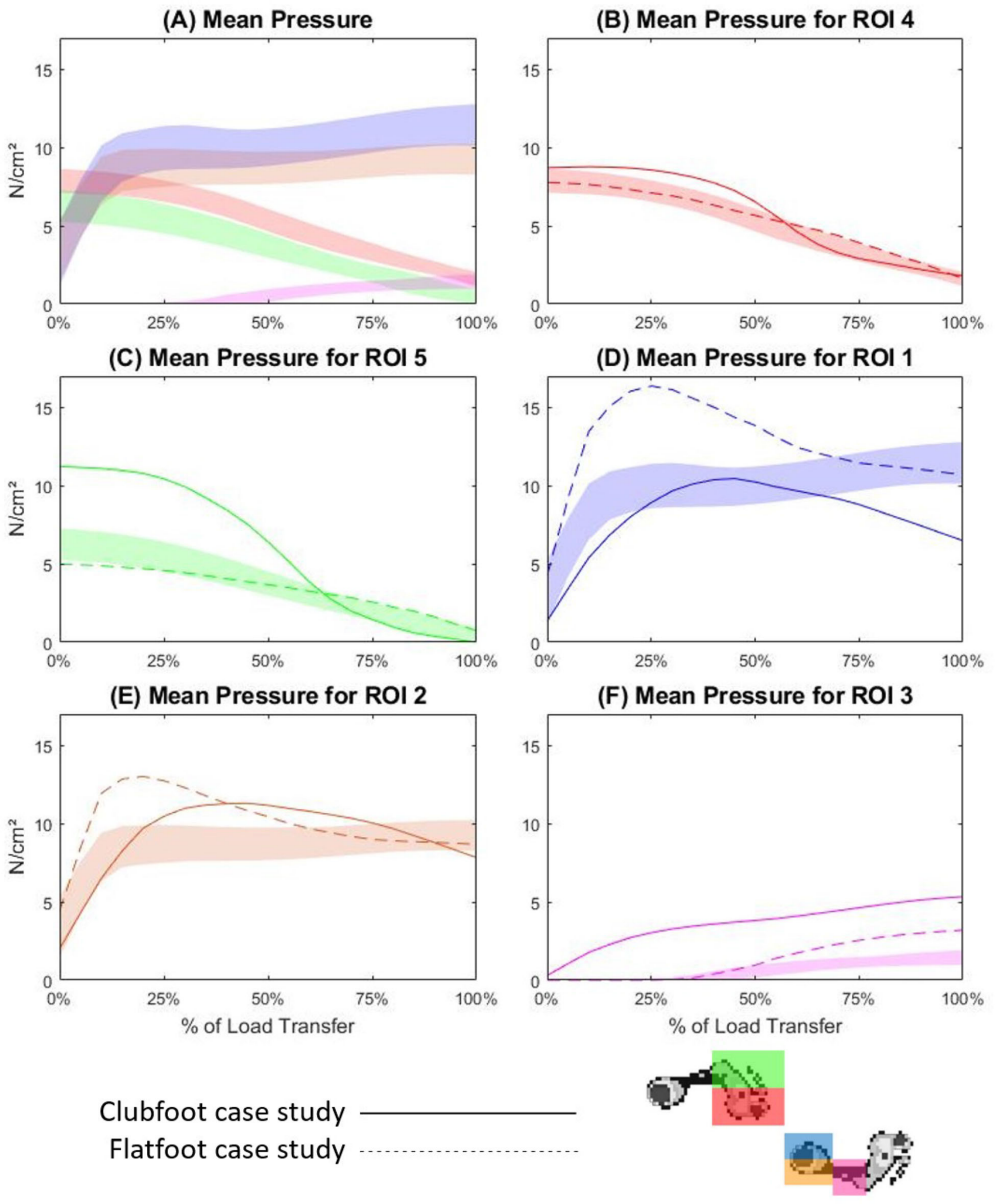
Mean pressure across the load transfer phase of gait for all regions (5**A**), ROI4 (5**B**), ROI5 (5**C**), ROI1 (5**D**), ROI2 (5**E**) and ROI3 (5**F**). The coloured band is representative of one standard deviation either side of the mean for the TD cohort, the solid line is the mean trace for case study one, and the dotted line is the mean trace for case study two

Mean pressure refers to the average pressures of all sensors within the ROI at each timepoint. At commencement of LT phase in the TD cohort, the largest mean pressures were recorded in ROI4 and ROI5 with averages of 7.88 N/cm^2^ and 6.29 N/cm^2^ respectively (Supplementary Table 1). Mean pressures then steadily declined across the remainder of the LT phase (Fig. 5A, B and C). Mean pressures for ROI1 and ROI2 showed a steep incline from an average of 3.32 N/cm^2^ at commencement of the LT phase to a mean of 8.12 N/cm^2^ at 10%, before plateauing across the rest of the LT phase (Fig. 5A, D and E). Initial mean pressures for ROI3 exceeded 1 N/cm^2^ at 55% of the LT phase and show a steady incline to reach a peak at 1.46 N/cm^2^ at 100% (Supplementary Table 1 and Fig. 5F). For case study one, the mean pressures for ROI4 were slightly increased compared to the TD band but followed a very similar pattern (Fig. 5B). Mean pressures for ROI5 were nearly double the TD band at commencement of the LT phase (Supplementary Table 1) and exhibited a steady incline until overlapping the TD band at 65% of the LT phase (Fig. 5C). Case study one mean pressure for ROI1 exhibited a skewed bell-shaped curve, overlapping the lower limits of the TD band between 25 to 65% of the LT phase, and peaking at 10.46 N/cm^2^ (Additional file 1 and Fig. 5D). The mean pressure curve for ROI2 exceeded the upper limit of the TD band, peaking at 11.30 N/cm^2^ at 45% of the LT phase (Supplementary Table 1 and Fig. 5E). Finally, mean pressure in ROI3 displayed a relatively steady increase across the entire LT phase, peaking at 5.35 N/cm^2^ at 100% (Supplementary Table 1 and Fig. 5F). For case study two, mean pressures in ROI4 and ROI5 closely resembled the TD patterns, overlapping the TD bands for 80 and 55% of the LT phase, respectively (Supplementary Table 1 and Fig. 5B and C). Case study two mean pressure in ROI1 exhibited a heavily skewed bell-shaped curve which exceeded the TD upper limits from 5 to 65% of the LT phase, peaking at 16.37 N/cm^2^ at 25% (Supplementary Table 1 and Fig. 5D). Mean pressure in ROI2 also exhibited a similar pattern, exceeding the TD band from 5 to 55% of the LT phase, peaking at 13.02 N/cm^2^ at 20% (Supplementary Table 1 and Fig. 5E). Finally, mean pressures in ROI3 commenced at 30% of LT, and increased above the TD band at 50% of the LT phase, peaking at 3.21 N/cm^2^ (Supplementary Table 1 and Fig. 5F).

## Discussion

The aim of this study was to provide reference pressure data, divided into anatomical regions, for a typically developed population across the LT phase of gait as a comparative tool for clinical populations. Data was also presented for two pathological case studies with abnormal joint morphology and joint mobility, and compared relative to the reference data to determine the clinical utility of examining the LT phase. The results indicated the importance of the LT phase of gait by highlighting the clear differences during this phase for the two case studies presented. For case study one, the clubfoot participant, there were clear differences between the leading and trailing foot for all variables, with the trailing foot appearing to affect foot pressures in the leading step. For case study two, the flatfoot case study, pressures in the trailing foot were consistent with TD children although the leading foot medial and lateral pressures were on average 1.4 times higher than TD children. The greatest differences seen in mid-foot pressures occurred following the LT phase.

### Typically developed population

For the TD population, our participants produced forces in the LT phase consistent with those presented in the literature [14, 16]. In agreement with previous findings, we observed increased values for the medial forefoot during push-off in the trailing step across all pressure and force parameters [14, 16]. In the leading step, we saw a balanced hindfoot, producing equal forces and pressures in the first 25% of LT, before a slight increase in pressure medially. It has been suggested in the literature that following a typically balanced heel strike, pressures progress laterally through the midfoot, with normal participants producing peak lateral-medial force indexes of approximately 1.6 during midstance (ie. increased lateral forces compared to medial) [37]. This slight medial shift in our TD participants may have been due to the transfer of load from the opposing limb originating from the medial side of the limb. After toe-off (Fig. 1A) there was a pressure shift laterally again during midstance. Midfoot contact was first seen at approximately 40% of the LT phase, which was indicative of a clear heel strike before flatfoot is achieved. Despite not being evident in the LT phase data, there was a medial shift in the forefoot across the second half of the stance phase (Fig. 1A).This is characteristic of typically developed gait [11, 12].

In the contact area graphs, total forefoot contact area was greater than total hindfoot contact area. This was to be expected given the anatomy of the plantar surface of the foot. As the same amount of force is being applied to a smaller contact area in the hindfoot, there was an increase in mean pressure for the hindfoot. The differences in peak pressures, from forefoot to hindfoot, were not as large. We suspect that this was due to the medial forefoot’s large contact area, as most of the pressure was concentrated over the first metatarsal and hallux, producing similar peak pressures to a region with a smaller contact area. It is important to note that contact areas were reported in absolute values. Overall, the TD pressures observed in our participant cohort was in agreement with previous findings across the entire footprint [14, 16].

### Case study one: clubfoot

The contact area and force graphs for the trailing foot revealed a lateral bias for case study one. Contact area for both medial and lateral regions in the trailing forefoot were slightly larger at the start of the LT phase, perhaps due to an increase in double-limb support duration (Table 1), and or the larger stature of this participant compared to the mean TD participant. Observation of the force graphs revealed that the clubfoot participant demonstrated increased lateral forefoot forces and reduced medial forces in the first 30% of the LT phase, which was due to hindfoot-tibia adduction, and forefoot-tibia inversion of the trailing foot (see Supplementary Figure 3). Lateral dominance was more pronounced in the mean pressure and peak pressure graphs, with peak pressures in case study one doubling those seen in the TD population. Further, increased peak pressures were evident medially, however these occurred under the second and third metatarsal head rather than the first metatarsal and hallux. These increased peak pressures occur only in the first 65% of the LT phase, before overlapping the TD participant band. Early peak pressures and decreased forces during LT resulted in decreased ankle power at push off (see Supplementary Figure 4).

The largest differences seen in the contact area graphs was in the lateral hindfoot, where peak contact area was two and a half times the magnitude of the typical band. We also observed an increase in the contact area of the medial hindfoot at 30% of the LT phase, likely due to increased inversion during swing (see Supplementary Figure 3) [30]. This would have also led to a large lateral contact area at initial contact, followed by an increased medial contact during loading. Unsurprisingly, the lateral contact area remained larger across the entire LT phase. Case study one also exhibited an earlier and larger contact area in the midfoot due to the combined kinematic impacts of the deformity at ankle and foot joints (i.e. hindfoot-tibia equinus and inversion; see Supplementary Figure 3), resulting in a loss of heel strike and flat foot at initial contact. There were also increases in force, mean pressure and peak pressure at the lateral hindfoot of the leading step compared to TD participants (Figs. 3E, 4E and 5E). In contrast with the contact area graphs, there was a decrease in force and mean pressure for the medial hindfoot (Figs. 3D and 5D). The pattern of the pressure curves was also different with a smooth inverted U shape peaking at an average of 45% of the LT phase observed for all graphs, again highlighting the lack of a prominent heel strike in the hindfoot. The unloading observed at the end of the LT phase for all variables is not seen in the TD bands for the hindfoot and is possibly a stabilising adaptation in the clubfoot participant [38]. Finally, the lateral hindfoot graphs for the clubfoot participant more closely resembled those for the medial hindfoot. We hypothesise that this is due to the large increase in contact area for this region.

### Case study two: flat foot

Case study two presented with increased velocity and a slightly increased percentage of time in double limb support. The forefoot of the trailing step for the case study two demonstrated a similar pattern for the majority of LT metrics with considerable overlap with the TD graphs. There was a tendency however, toward medial forefoot bias for contact area and force. Indeed, typical profiles are generally observed at the forefoot during double support in flat foot patients [39]. The largest differences for all reported variables was during the LT phase at the level of the medial hindfoot, where significant increases in force (23% increase), mean pressure (28% increase) and peak pressure (66% increase) were evident. Increased hindfoot-tibia eversion seen at heel strike in the foot model kinematics verified these force and pressure findings (see Supplementary Figure 5). Increased forces were also seen in the lateral hindfoot and midfoot, with midfoot forces continuing to increase after the completion of LT (Fig. 1B). This was supported by the hindfoot-tibia kinematics, which revealed greater eversion compared to the TD band following the LT phase (see Supplementary Figure 5). This may have been due to insufficient contributions from the intrinsic foot muscles responsible for the storage and return of elastic energy within the arch [40] or structural changes such as calcaneal inversion and talar adduction [31]. Midfoot forces could also continue to increase across stance as a consequence of the medial forefoot bias seen during the LT phase. Differences in mean and peak pressure curves compared to force showed a large peak at approximately 25% of LT followed by a gradual decline ending within the TD band for the mean pressure and just above the TD band for peak pressure. This provides support to the premise of a ‘heavy’ heel strike in case study two compared to TD participants, resulting in a slightly increased power absorption at the ankle and knee (Supplementary Figure 6). Compared to contact area profiles, pressures begin to normalise as contact area increases above the TD band. The lateral hindfoot followed a similar pattern in the peak and mean pressures, but to a lesser extent. This medial shift is supported by centre of pressure (COP) data that has been reported in the literature [37, 41, 42]. Specifically, COP lines in previous studies have reported to be medially deviated in flat foot participants [39, 41], and as such we would expect to see a medial shift across the whole foot. Typically, increases in force have been documented in the mid- and forefoot of flatfoot participants during stance, but not in the hindfoot [43]. A focus on the LT phase highlights the increases in medial force and pressure in the hindfoot which may have not been presented previously.

Finally, the midfoot of the flatfoot case study followed a similar pattern to the TD population, as contact is not observed until 30% of the LT phase. This means that case study two demonstrates a defined heel strike before foot flat occurs. Once foot flat occurred however, a steeper incline across all four variables compared to TD participants was observed. The greatest increases were seen in contact area and force. Not only was a larger portion of the plantar surface in contact with the ground, but force was also being applied to it. Peak pressures showed the smallest differences to the TD cohort, suggesting that the increased forces seen are evenly dispersed across the plantar area. During double limb support the opposing limb was still supporting a portion of the load, resulting in the midfoot being less deformed and therefore producing lower pressures. During single limb support, however, force in the midfoot continued to increase, peaking at 45% of the leading foot’s stance phase (Fig. 1C). We suspect that whilst the greatest midfoot differences in the flatfoot case study occurred after termination of the LT phase, it may have been due to the hindfoot-tibia inversion present during LT which could be corrected with an orthotic.

The pressure reported for the flat foot case study is in good agreement with kinematic data presented in Supplementary figure 5 and in the literature [36, 44], suggesting that plantar loads analysis that incorporates anatomical landmark projections might be sufficient for clinical interpretation when full lower limb kinematics is not feasible. Access to both 3D motion capture systems and plantar load devices may be confined to hospital based gait laboratories and clinical research facilities. Nonetheless, our findings suggest that collection of a minimal marker set and plantar loads may allow for a more economical option for clinical evaluation of foot motion when a fully equipped laboratory is not available. It’s important to acknowledge that our TD cohort was relatively small for a normative database, with a large age range of participants at various stages of development and gait maturity. Additionally, the case studies do not necessarily represent the range of foot impairments seen in CTEV and flatfoot patients. Nonetheless, our data does provide evidence of the importance of examining the LT phase in clinical management. Future studies should investigate the impact of gait maturation on regional pressure and force metrics, using a larger dataset that can be evaluated with cluster analysis, to determine whether age specific normative reference data is indicated. It would also be beneficial to produce complete data sets for various clinical populations. It is likely not feasible to review all force, area and pressure profiles in focus on the LT phase in a clinical review. Keijsers and colleagues [45] report high correlations between reported peak pressure and mean pressure variables in a typical population, suggesting reporting fewer parameters is sufficient. We therefore recommend that normalised force and peak pressures should be the first point of reference. These variables show how force is transferred between regions and highlight areas of concern for pressure sores and ulceration. In summary our findings highlight (i) the importance of isolating data collected during double limb support and (ii) examining the LT phase has the potential to provide clinically meaningful information for intervention planning.

## Supplementary Information

**Additional file 1: Supplementary Table S1.** Peak variable outcomes for all regions of interest. Peak contact area, force, peak pressure and mean pressure for the typically developed (TD) cohort, the clubfoot (CTEV) case study and the flatfoot (FF) case study for all regions of interest during the load transfer (LT) phase.

**Additional file 2: Supplementary Figure S1.** Individual normalised force traces. Individualised normalised force traces for participants under 7 (blue), under 12.5 (red) and over 12.5 (green) for each region of interest during the load transfer phase.

**Additional file 3: Supplementary Figure S2.** Individual peak pressure traces. Individualised peak pressure traces for participants under 7 (blue), under 12.5 (red) and over 12.5 (green) for each region of interest during the load transfer phase.

**Additional file 4: Supplementary Figure S3.** Oxford foot model kinematics for the clubfoot case study. Oxford foot model kinematics for the right (green) and left (red) limbs for the clubfoot case study as compared to a typically developed population (grey) across the gait cycle as presented at the QCMAS. For this participant, the right side is most affected. The asterisk in graph titles denotes where figures axes have needed to be adjusted based on participant outputs.

**Additional file 5: Supplementary Figure S4.** Kinetics for the clubfoot case study. Kinetics for the right (green) and left (red) limbs for the clubfoot case study as compared to a typically developed population (grey) across the gait cycle as presented at the QCMAS. For this participant, the right side is most affected.

**Additional file 6: Supplementary Figure S5.** Oxford foot model kinematics for the flatfoot case study. Oxford foot model kinematics for the right (green) and left (red) limbs for the flatfoot case study as compared to a typically developed population (grey) across the gait cycle as presented at the QCMAS. For this participant, the left side is most affected.

**Additional file 7: Supplementary Figure S6.** Kinetics for the flatfoot case study. Kinetics for the right (green) and left (red) limbs flatfoot case study as compared to a typically developed population (grey) across the gait cycle as presented at the QCMAS. For this participant, the left side is most affected.

## Data Availability

The datasets used and/or analysed during the current study are available from the corresponding author on reasonable request.

## Abbreviations

QCMAS: Queensland Children’s Motion Analysis Service
CP: Cerebral Palsy
CTEV: Congenital Talipes Equinovarus or Clubfoot
ROI: Region of Interest
GRF: Ground Reaction Force
EMG: Electromyography
TD: Typically Developed
PiG: Plug in Gait
OFM: Oxford Foot Model
LT: Load Transfer
LR: Left to right transfer
RL: Right to left transfer
m: Metres
m/s: Metres per second
