# Supplementary material for: Plantar load transfer in children: a descriptive study with two pathological case studies
Source: BMC Musculoskelet Disord. 2021 Jun 7;22:521. doi: 10.1186/s12891-021-04364-9 (PMC8185932; doi:10.1186/s12891-021-04364-9)
Supplement: Supplementary file 1 — Additional file 1: Supplementary Table S1. Peak variable outcomes for all regions of interest. Peak contact area, force, peak pressure and mean pressure for the typically developed (TD) cohort, the clubfoot (CTEV) case study and the flatfoot (FF) case study for all regions of interest during the load transfer (LT) phase. [file 12891_2021_4364_MOESM1_ESM.docx]

|  | | **Contact Area** | | | | | **Force** | | | | | **Peak Pressure** | | | | | **Mean Pressure** | | | | |
| --- | --- | --- | --- | --- | --- | --- | --- | --- | --- | --- | --- | --- | --- | --- | --- | --- | --- | --- | --- | --- | --- |
|  |  | **ROI4** | **ROI5** | **ROI1** | **ROI2** | **ROI3** | **ROI4** | **ROI5** | **ROI1** | **ROI2** | **ROI3** | **ROI4** | **ROI5** | **ROI1** | **ROI2** | **ROI3** | **ROI4** | **ROI5** | **ROI1** | **ROI2** | **ROI3** |
| **MAXIMUM** | **TD Upper Limit** | 27.59 | 19.55 | 12.86 | 13.48 | 4.38 | 72.95 | 43.10 | 50.45 | 42.62 | 4.29 | 33.07 | 21.48 | 32.69 | 28.52 | 3.03 | 8.65 | 7.29 | 12.79 | 10.24 | 1.93 |
|  | **TD Lower Limit** | 22.26 | 13.49 | 10.37 | 10.77 | 2.43 | 54.04 | 25.10 | 35.07 | 30.56 | 1.90 | 21.34 | 14.82 | 24.21 | 21.35 | 1.51 | 7.10 | 5.29 | 10.13 | 8.26 | 0.99 |
|  | **% of LT** | 0 | 0 | 100 | 100 | 100 | 0 | 0 | 100 | 100 | 100 | 0 | 0 | 100 | 100 | 100 | 0 | 0 | 100 | 100 | 100 |
|  | **CTEV Case Study** | 29.74 | 23.55 | 16.32 | 24.93 | 14.83 | 45.39 | 45.91 | 28.46 | 45.63 | 13.82 | 34.23 | 36.83 | 22.02 | 21.43 | 9.81 | 8.78 | 11.22 | 10.46 | 11.31 | 5.35 |
|  | **% of LT** | 0 | 0 | 60 | 20 | 100 | 0 | 0 | 50 | 30 | 100 | 20 | 0 | 45 | 40 | 100 | 10 | 0 | 45 | 45 | 100 |
|  | **FF Case Study** | 26.90 | 15.93 | 16.94 | 15.53 | 11.19 | 69.09 | 26.26 | 62.09 | 44.75 | 14.39 | 22.78 | 15.36 | 54.50 | 45.68 | 5.67 | 7.78 | 5.00 | 16.37 | 13.03 | 3.21 |
|  | **% of LT** | 0 | 0 | 85 | 100 | 100 | 0 | 0 | 70 | 100 | 100 | 15 | 0 | 20 | 20 | 100 | 0 | 0 | 25 | 20 | 100 |
| **MINIMUM** | **TD Upper Limit** | 1.89 | 0.42 | 5.27 | 6.30 | 0.00 | 1.31 | 0.23 | 9.95 | 13.19 | 0.00 | 2.95 | 1.07 | 12.23 | 12.84 | 0.00 | 2.05 | 0.95 | 5.29 | 5.36 | 0.00 |
|  | **TD Lower Limit** | 0.37 | 0.00 | 0.85 | 1.41 | 0.00 | 0.18 | 0.00 | 0.05 | 0.33 | 0.00 | 1.21 | 0.00 | 1.06 | 1.43 | 0.00 | 1.13 | 0.00 | 1.15 | 1.45 | 0.00 |
|  | **% of LT** | 100 | 100 | 0 | 0 | 0 | 100 | 100 | 0 | 0 | 0 | 100 | 100 | 0 | 0 | 0 | 100 | 100 | 0 | 0 | 0 |
|  | **CTEV Case Study** | 1.48 | 0.00 | 1.94 | 7.51 | 0.65 | 0.52 | 0.00 | 1.06 | 4.04 | 0.18 | 2.48 | 0.00 | 2.09 | 2.98 | 0.38 | 1.80 | 0.00 | 1.38 | 2.02 | 0.30 |
|  | **% of LT** | 100 | 100 | 0 | 0 | 0 | 100 | 100 | 0 | 0 | 0 | 100 | 100 | 0 | 0 | 0 | 100 | 100 | 0 | 0 | 0 |
|  | **FF Case Study** | 1.17 | 0.22 | 3.78 | 3.63 | 0.00 | 0.76 | 0.11 | 7.24 | 8.49 | 0.00 | 2.09 | 0.83 | 10.45 | 11.26 | 0.00 | 1.63 | 0.74 | 4.28 | 4.45 | 0.00 |
|  | **% of LT** | 100 | 100 | 0 | 0 | 0 | 100 | 100 | 0 | 0 | 0 | 100 | 100 | 0 | 0 | 0 | 100 | 100 | 0 | 0 | 0 |
| **% OF MEAN WITHIN TD BANDS** | **CTEV Case Study** | 55 | 20 | 15 | 0 | 0 | 10 | 35 | 25 | 10 | 0 | 40 | 15 | 20 | 35 | 0 | 40 | 25 | 45 | 40 | 0 |
|  | **FF Case Study** | 100 | 100 | 25 | 40 | 35 | 90 | 100 | 5 | 5 | 35 | 65 | 75 | 5 | 5 | 40 | 80 | 55 | 35 | 45 | 45 |

Supplementary Table 1. Peak variable outcomes for all regions of interest.

Peak contact area, force, peak pressure and mean pressure for the typically developed (TD) cohort, the clubfoot (CTEV) case study and the flatfoot (FF) case study for all regions of interest during the load transfer (LT) phase.
