# Supplementary figures and images for: Plantar load transfer in children: a descriptive study with two pathological case studies
Source: BMC Musculoskelet Disord. 2021 Jun 7;22:521. doi: 10.1186/s12891-021-04364-9 (PMC8185932; doi:10.1186/s12891-021-04364-9)

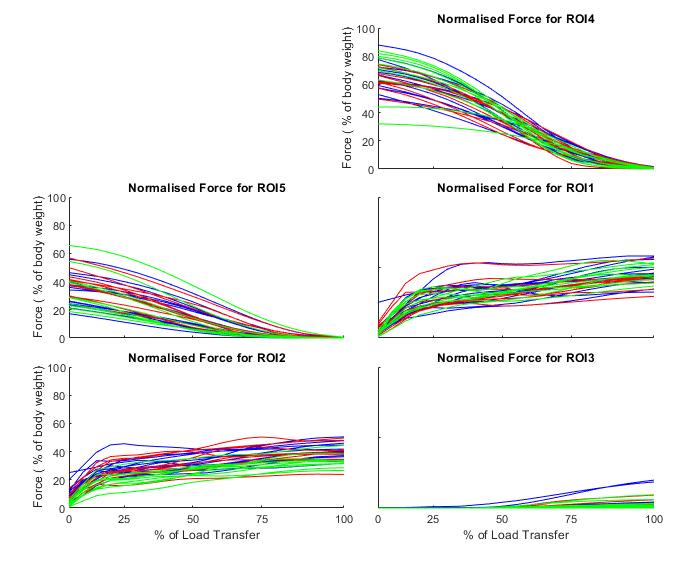

Supplement: Supplementary file 2 — Additional file 2: Supplementary Figure S1. Individual normalised force traces. Individualised normalised force traces for participants under 7 (blue), under 12.5 (red) and over 12.5 (green) for each region of interest during the load transfer phase. [file 12891_2021_4364_MOESM2_ESM.jpg]

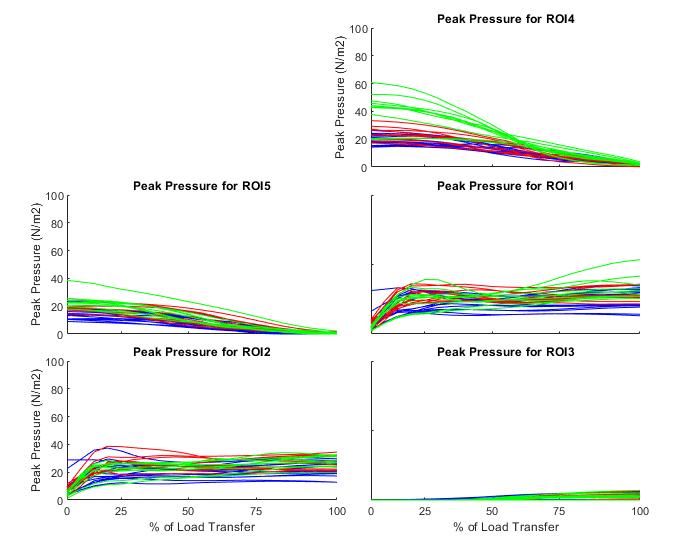

Supplement: Supplementary file 3 — Additional file 3: Supplementary Figure S2. Individual peak pressure traces. Individualised peak pressure traces for participants under 7 (blue), under 12.5 (red) and over 12.5 (green) for each region of interest during the load transfer phase. [file 12891_2021_4364_MOESM3_ESM.jpg]

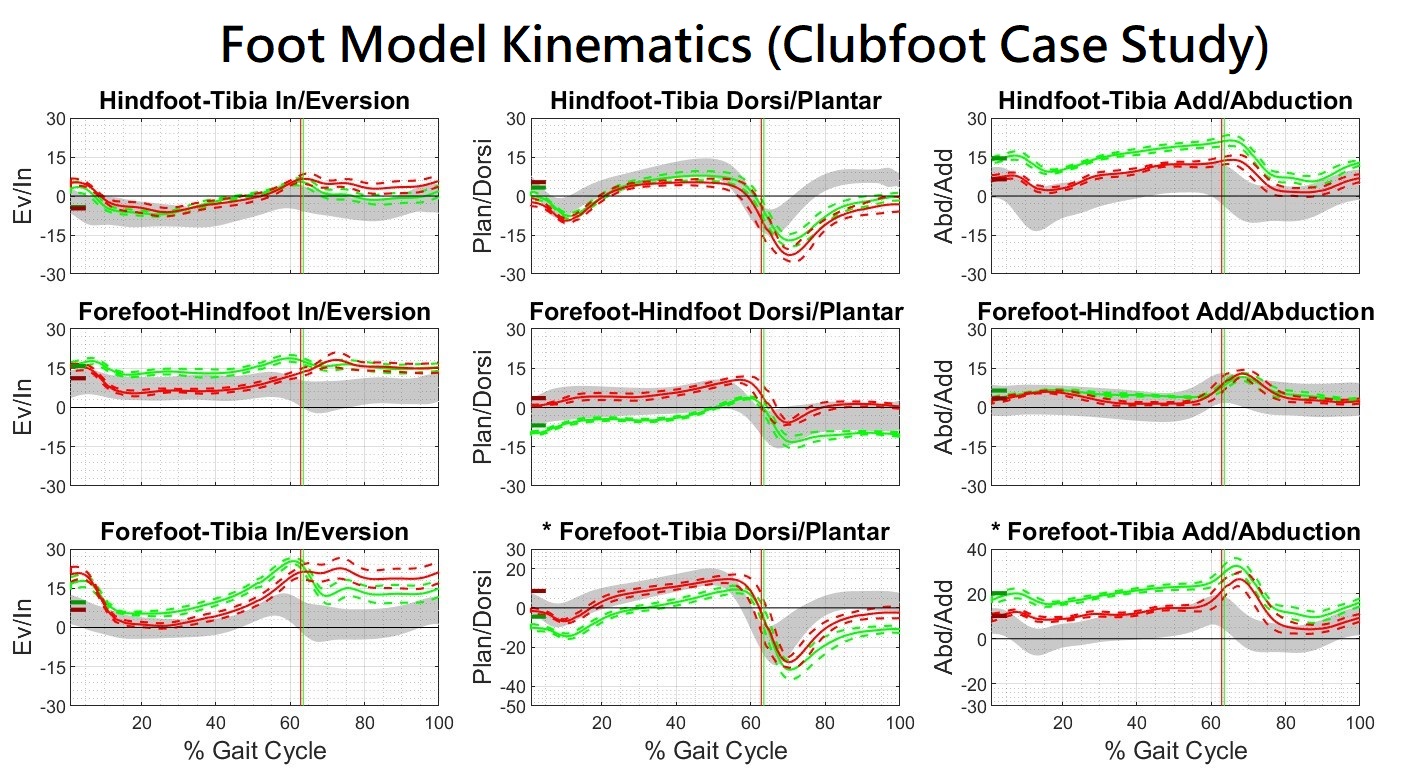

Supplement: Supplementary file 4 — Additional file 4: Supplementary Figure S3. Oxford foot model kinematics for the clubfoot case study. Oxford foot model kinematics for the right (green) and left (red) limbs for the clubfoot case study as compared to a typically developed population (grey) across the gait cycle as presented at the QCMAS. For this participant, the right side is most affected. The asterisk in graph titles denotes where figures axes have needed to be adjusted based on participant outputs. [file 12891_2021_4364_MOESM4_ESM.jpg]

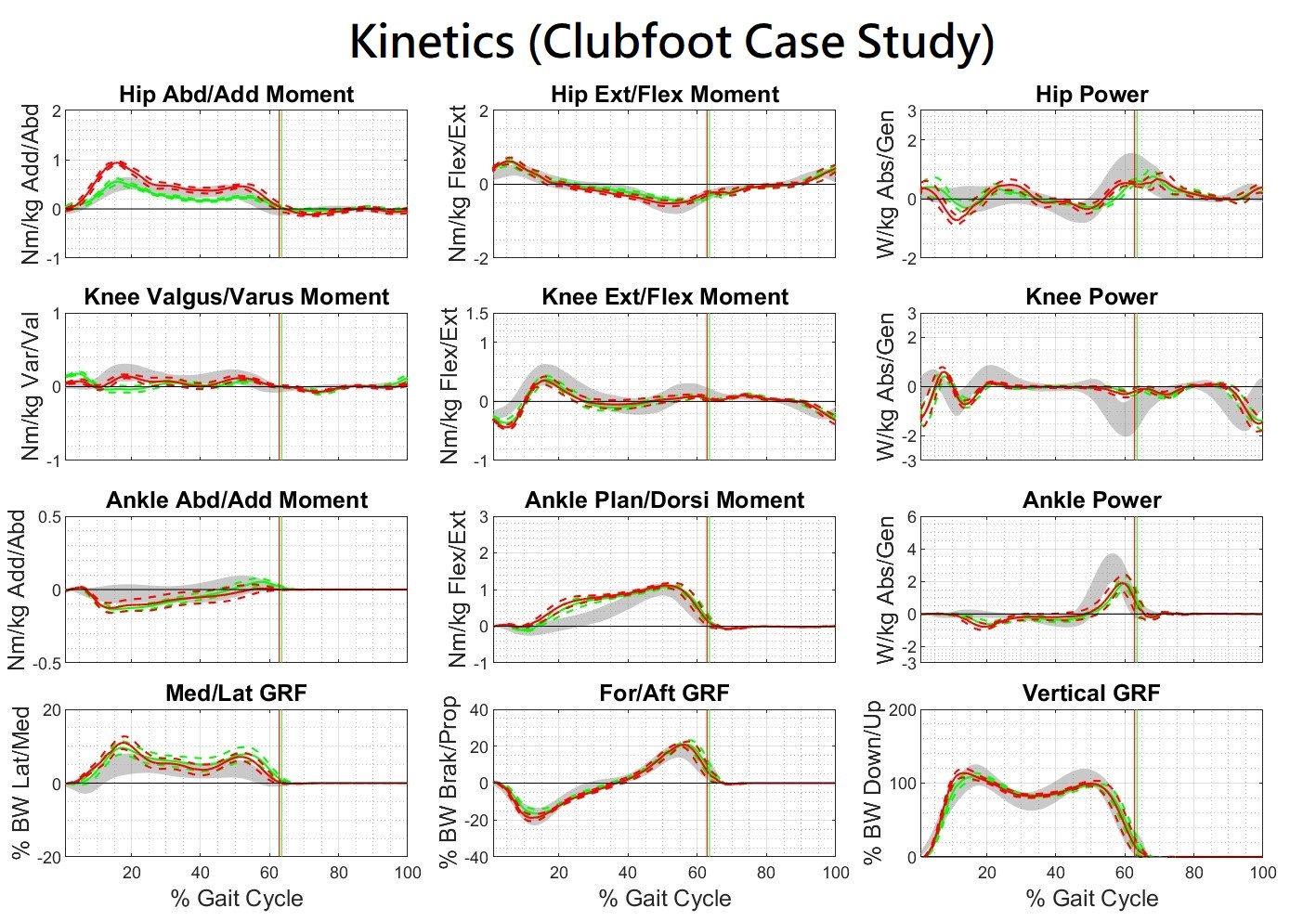

Supplement: Supplementary file 5 — Additional file 5: Supplementary Figure S4. Kinetics for the clubfoot case study. Kinetics for the right (green) and left (red) limbs for the clubfoot case study as compared to a typically developed population (grey) across the gait cycle as presented at the QCMAS. For this participant, the right side is most affected. [file 12891_2021_4364_MOESM5_ESM.jpg]

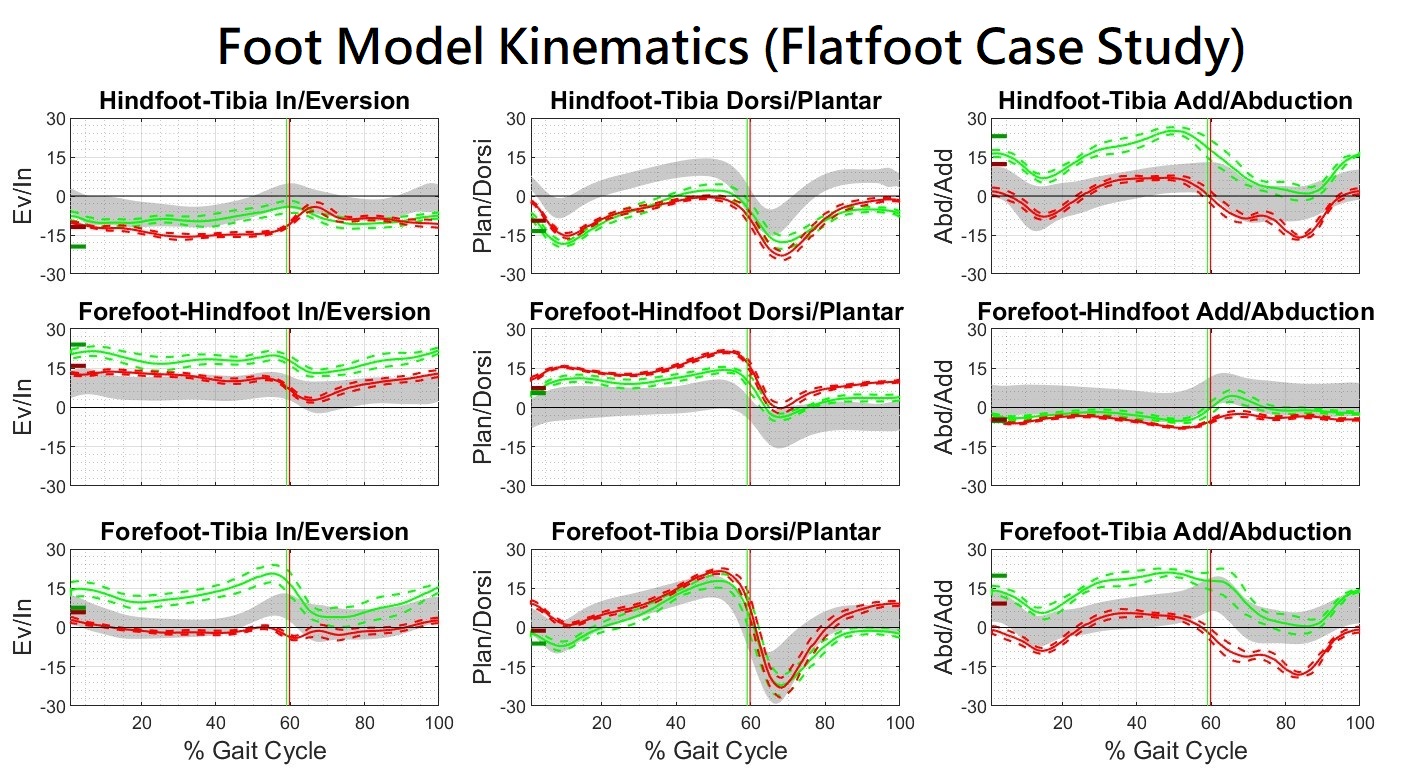

Supplement: Supplementary file 6 — Additional file 6: Supplementary Figure S5. Oxford foot model kinematics for the flatfoot case study. Oxford foot model kinematics for the right (green) and left (red) limbs for the flatfoot case study as compared to a typically developed population (grey) across the gait cycle as presented at the QCMAS. For this participant, the left side is most affected. [file 12891_2021_4364_MOESM6_ESM.jpg]

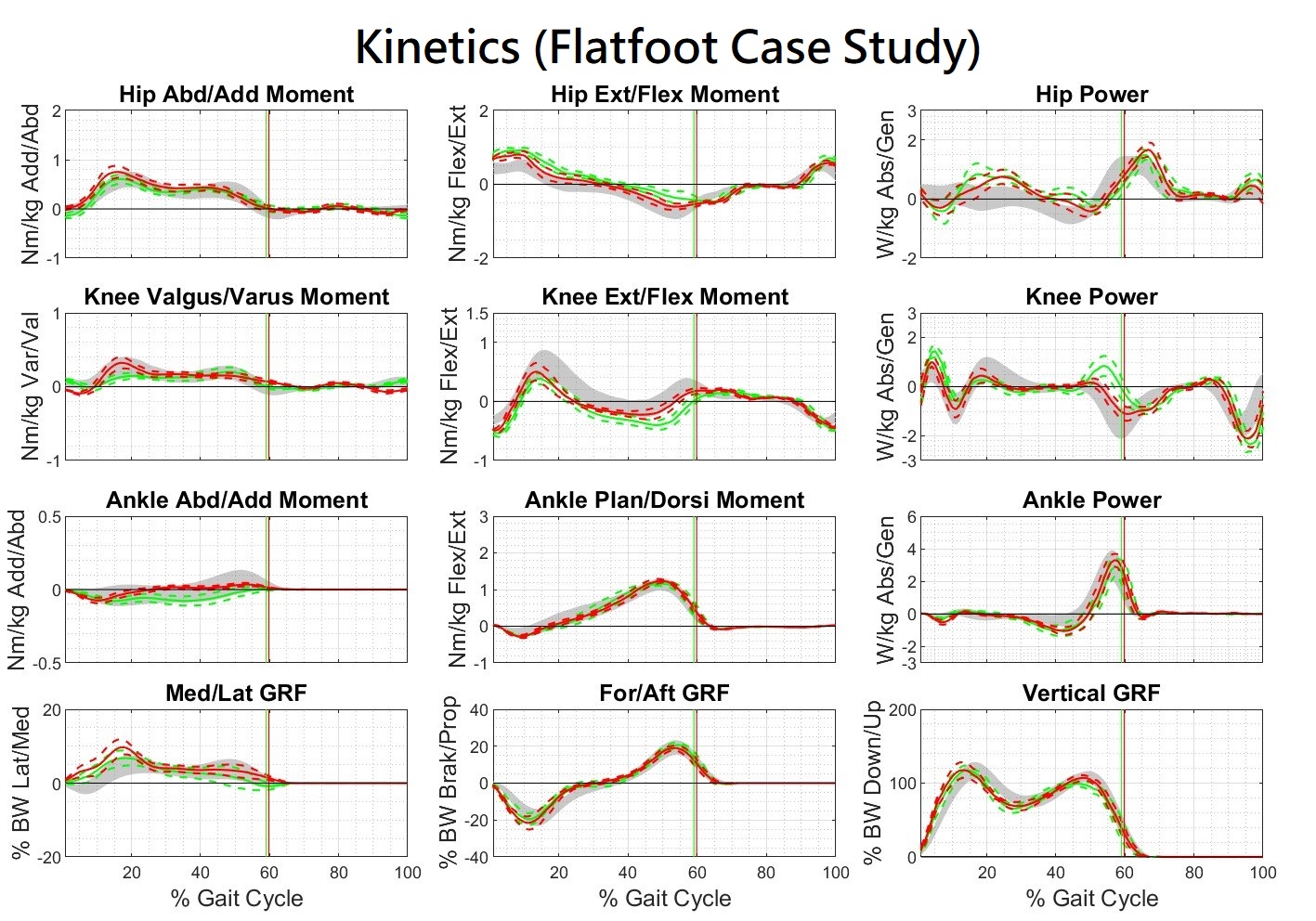

Supplement: Supplementary file 7 — Additional file 7: Supplementary Figure S6. Kinetics for the flatfoot case study. Kinetics for the right (green) and left (red) limbs flatfoot case study as compared to a typically developed population (grey) across the gait cycle as presented at the QCMAS. For this participant, the left side is most affected. [file 12891_2021_4364_MOESM7_ESM.jpg]
